# Comparative Outcomes of Total Versus Partial Pericardiectomy in Constrictive Pericarditis: A Two-Decade Single-Centre Experience

**DOI:** 10.1093/icvts/ivag147

**Published:** 2026-05-22

**Authors:** Karim R Moawad, Mostafa M Elbadry Mohamed, Mohamed Abdelkhalik, Hazem Aljasem, Jason Kho, Dimitrios Pousious, Sunil K Ohri

**Affiliations:** Department of Cardiac Surgery, University Hospital Southampton NHS Foundation Trust, Southampton, SO16 6YD, United Kingdom; Department of Cardiac Surgery, University Hospital Southampton NHS Foundation Trust, Southampton, SO16 6YD, United Kingdom; Department of Cardiothoracic Surgery, Heart Hospital, Assiut University, Assiut, Egypt; Department of Cardiac Surgery, University Hospital Southampton NHS Foundation Trust, Southampton, SO16 6YD, United Kingdom; Department of Cardiac Surgery, University Hospital Southampton NHS Foundation Trust, Southampton, SO16 6YD, United Kingdom; Department of Cardiac Surgery, University Hospital Southampton NHS Foundation Trust, Southampton, SO16 6YD, United Kingdom; Department of Cardiac Surgery, University Hospital Southampton NHS Foundation Trust, Southampton, SO16 6YD, United Kingdom; Department of Cardiac Surgery, University Hospital Southampton NHS Foundation Trust, Southampton, SO16 6YD, United Kingdom

**Keywords:** constrictive pericarditis, pericardiectomy, total pericardiectomy, partial pericardiectomy, right ventricular function, survival

## Abstract

**Objectives:**

The optimal extent of pericardial resection in constrictive pericarditis (CP) remains a subject of surgical debate. This study compared perioperative outcomes, postoperative right ventricular (RV) function, functional status, and long-term survival between total and partial pericardiectomy in a contemporaneous single-centre cohort.

**Methods:**

A retrospective analysis was performed on 102 consecutive patients undergoing pericardiectomy for CP at a single tertiary cardiac surgical centre between 2005 and 2025. Patients were stratified by operative extent into total pericardiectomy (*n* = 89) and partial pericardiectomy (*n* = 13). Preoperative, operative, and postoperative variables were compared. Long-term survival was assessed by Kaplan-Meier analysis with log-rank testing.

**Results:**

Patients in the partial group were older (67 ± 11 vs 63 ± 12 years; *P* = .240) with higher operative risk by EuroSCORE II (7.2 ± 4.6 vs 4.9 ± 2.7; *P* = .010) and logistic EuroSCORE (14.5 ± 19.3 vs 5.6 ± 6.8; *P* <0.001). Urgent/emergency cases were more frequent in the partial group (61.5% urgent, 15.4% emergency vs 43.8% and 4.5%). Preoperative NYHA III-IV distribution was comparable (51.9% total vs 46.2% partial), as was preoperative RV function (*P* = .353).

**Conclusions:**

Despite greater operative complexity, total pericardiectomy confers significantly superior long-term survival and functional recovery compared with partial resection. These data support total pericardiectomy as the standard of care for durable relief of CP, with appropriately selected use of cardiopulmonary bypass to facilitate complete biventricular decortication.

## Graphical Abstract

**Figure:**
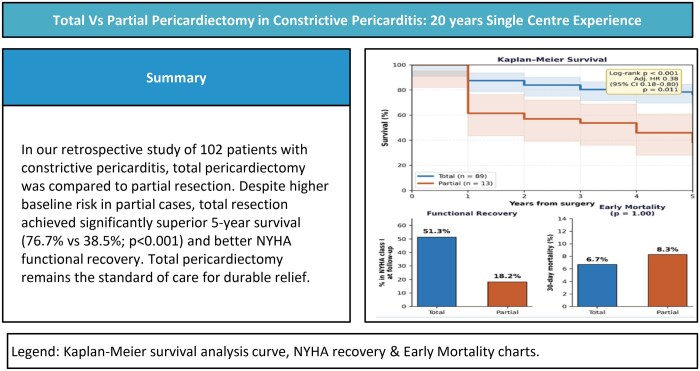

## INTRODUCTION

The pericardium is a multilayered fibroelastic sac encasing the heart, serving roles as a mechanical barrier, immunomodulator, and vasomotor coordinator. Under normal physiology it contains 10-50 mL of serous fluid.^1^ Constrictive pericarditis (CP) is a debilitating condition with a 3:1 male predominance in which chronic inflammation leads to pericardial fibrosis, thickening, and—in a substantial proportion of cases—calcification, resulting in progressive impairment of diastolic ventricular filling and, ultimately, biventricular failure.^2^

In contemporary Western series idiopathic/viral disease predominates (42%-49%), followed by postcardiac surgery (11%-37%) and postradiation pericarditis (9%-31%)^3^^,^^4^; tuberculous CP remains important in endemic regions.^5^ Patients typically present with exertional dyspnoea and fatigue, progressing to right heart failure with raised venous pressure, oedema, and hepatic congestion.^2^ Diagnosis integrates clinical features with multimodality imaging. Echocardiography with tissue Doppler (annulus paradoxus; respiratory variation in mitral E-velocity) distinguishes CP from restrictive cardiomyopathy.^6^ CT and CMR characterize pericardial thickening, calcification, and myocardial involvement^7^; in equivocal cases, invasive catheterization confirms the diagnosis via the square-root sign and ventricular discordance.^8^ Pericardiectomy is the only definitive treatment for established CP, with operative mortality of 5%-10%.^9^^,^^10^ The optimal strategy—extent of resection and selective cardiopulmonary bypass (CPB)—remains debated.^11^ Total pericardiectomy (phrenic-to-phrenic with biventricular decortication^3^) offers complete relief of constriction but is technically demanding and often requires CPB.^12^ Partial resection is simpler but risks residual constriction and organ congestion,^13^ and has been identified as an independent predictor of late mortality with a 4.5-fold higher death risk.^13^

Evidence comparing contemporary outcomes of total versus partial pericardiectomy from UK centres is limited. This study reports a 2-decade single-centre experience at University Hospital Southampton NHS Foundation Trust, comparing perioperative morbidity and mortality, postoperative right ventricular (RV) function and functional capacity, and long-term survival between the 2 surgical strategies, contextualized against the existing international literature.

## METHODS

### Study design and patient population

**Figure 1.**
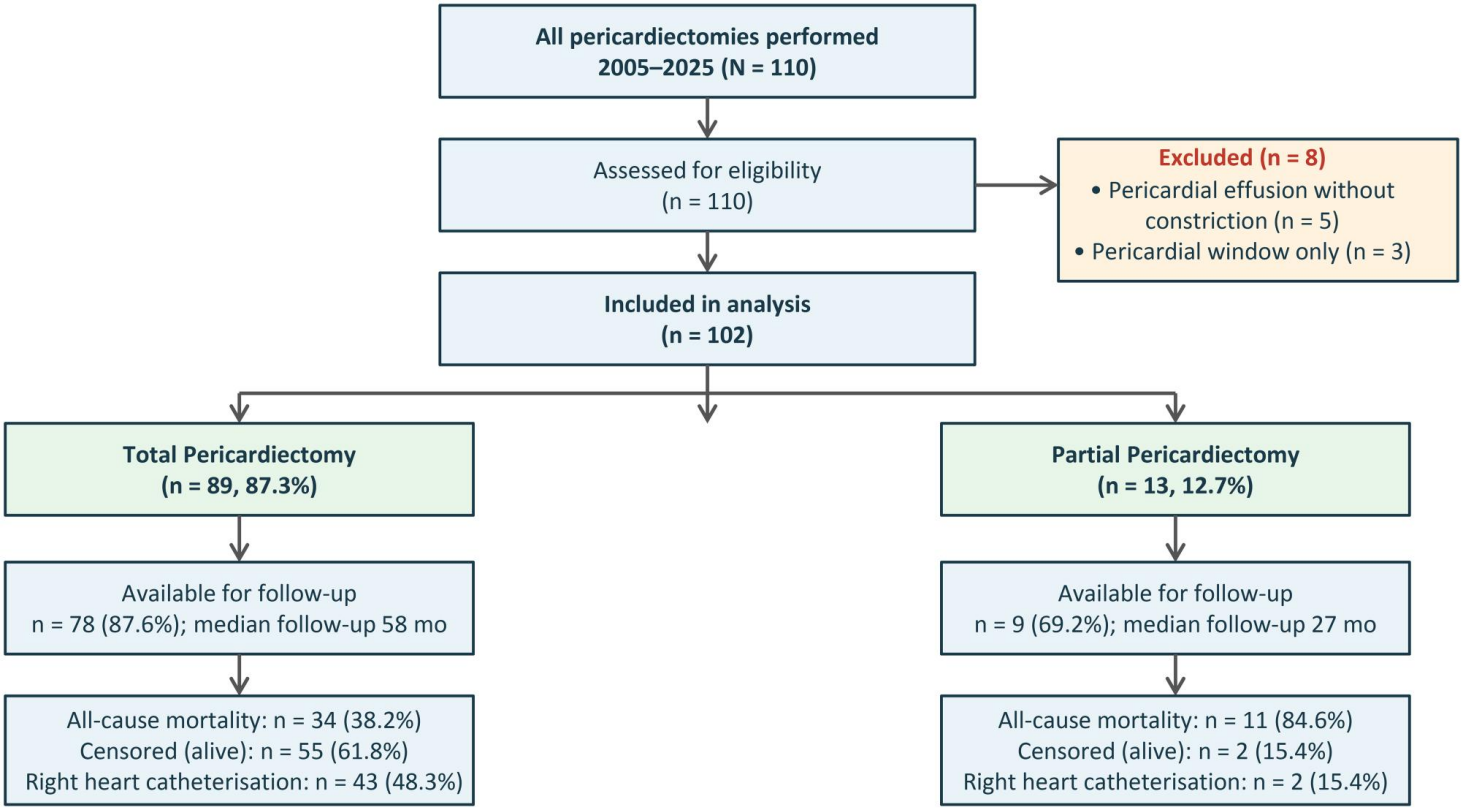
Patient Flow Diagram: Screening, Exclusions, Group Allocation, Follow-Up Availability, and Outcomes (2005-2025)

A retrospective analysis was performed of a prospectively maintained institutional cardiac surgical database. All consecutive patients undergoing pericardiectomy for CP at University Hospital Southampton between January 2005 and December 2025 were eligible. CP was confirmed by intraoperative findings in all cases; patients undergoing pericardial window procedures or pericardiectomy for pericardial effusion without constriction were excluded. A patient flow diagram is presented as **Figure 1**. The study was conducted in accordance with the Declaration of Helsinki. Institutional governance approval was obtained from the University Hospital Southampton NHS Foundation Trust. The institutional surgical database used for this study is prospectively maintained and monitored under institutional oversight in accordance with the principles of the WMA Declaration of Taipei regarding the ethical collection and storage of health data. Individual patient consent was waived due to the retrospective, observational nature of the study.

Patients were stratified into 2 groups based on operative report documentation of resection extent: total pericardiectomy (defined as excision from the right to left phrenic nerve, encompassing both ventricular free walls, the diaphragmatic surface, and the posterior pericardium to the maximum extent technically feasible; *n* = 89) and partial pericardiectomy (defined as intentionally limited resection falling short of complete biventricular decortication; *n* = 13). In all cases, including those undergoing concomitant cardiac surgery, CP was confirmed as the primary or co-primary indication for surgery based on operative report documentation and preoperative investigation; no patient was included in whom pericardiectomy represented an incidental or secondary procedure performed solely in the context of another dominant cardiac pathology.

### Data collection and variables

Preoperative data extracted included: patient demographics (age, sex, body mass index), operative urgency (elective, urgent, emergency), New York Heart Association (NYHA) functional classification, Canadian Cardiovascular Society (CCS) angina class, preoperative RV function on transthoracic echocardiography (categorized as good, mildly impaired, moderately impaired, or severely impaired), left ventricular ejection fraction (LVEF) category, tricuspid regurgitation (TR) grade, and comorbidity burden (diabetes mellitus, hypertension, pulmonary disease, neurological disease). Operative risk was quantified using both EuroSCORE II and logistic EuroSCORE. Where performed, right heart catheterization (RHC) data were recorded, including pulmonary artery systolic pressure and the presence of haemodynamic criteria for CP; the proportion of patients undergoing RHC is reported in results section.

Operative variables recorded included: use and duration of CPB, aortic cross-clamp time, use of intra-aortic balloon pump (IABP), and concomitant procedures (CABG, valve surgery). Postoperative outcomes included: ITU and total hospital length of stay, major morbidity events (deep sternal wound infection, return to theatre, neurological dysfunction, haemofiltration/dialysis, low cardiac output syndrome, RV failure, respiratory failure, hepatic dysfunction), and early mortality (defined as all-cause mortality within 30 days of surgery or during the index hospital admission). Follow-up data on NYHA class, RV function, and vital status were collected from outpatient clinic records and national death registries.

### Statistical analysis

Continuous variables are presented as mean ± SD (normally distributed data) or median (interquartile range) for non-normally distributed data; normality was assessed using the Shapiro-Wilk test. Between-group comparisons were performed using the independent-samples Student’s *t*-test or the Mann-Whitney *U*-test as appropriate. Categorical variables are reported as frequency (percentage) and compared using the chi-squared test or Fisher’s exact test as appropriate. Changes in ordinal categorical variables (RV function, NYHA class) between preoperative and follow-up assessments were assessed using the chi-squared test for trend. Long-term survival was estimated by the Kaplan-Meier method and compared between groups using the log-rank test. Cox proportional hazards regression estimated crude and adjusted hazard ratios (HR) with 95% CIs, adjusted for age, sex, and EuroSCORE II. All analyses were performed in R (version 4.3.1; R Foundation for Statistical Computing, Vienna, Austria). A 2-tailed *P-*value <.05 was considered statistically significant.

## RESULTS

### Baseline characteristics and operative risk profile

Between January 2005 and December 2025, 102 patients (73 male [71.6%], 29 female [28.4%]; mean age 64 ± 12 years) underwent pericardiectomy for CP at our institution. Of these, 89 (87.3%) underwent total and 13 (12.7%) partial pericardiectomy. Full patient characteristics are present in **Table  1**.

**Table 1.** Patient Characteristics, Operative Data, and Postoperative Outcomes

	All (*N* = 102)	Partial (*N* = 13)	Total (*N* = 89)	*P*-value
Demographics				
Age, years	64 ± 12	67 ± 11	63 ± 12	.24
Gender, female	29 (28.4%)	3 (23.1%)	26 (29.2%)	.90
BMI	28 ± 6	29 ± 5	28 ± 6	.70
EuroSCORE II	5.2 ± 3.1	7.2 ± 4.6	4.9 ± 2.7	.01
Logistic EuroSCORE	6.7 ± 9.6	14.5 ± 19.3	5.6 ± 6.8	<.01
Operative urgency				
Elective	49 (48.0%)	3 (23.1%)	46 (51.7%)	
Urgent	47 (46.1%)	8 (61.5%)	39 (43.8%)	
Emergency	6 (5.9%)	2 (15.4%)	4 (4.5%)	
Preoperative comorbidities				
CCS Angina Class				
I	58 (70.7%)	5 (41.7%)	53 (75.7%)	
II	12 (14.6%)	3 (25.0%)	9 (12.9%)	
III	9 (11.0%)	3 (25.0%)	6 (8.6%)	
IV	3 (3.7%)	1 (8.3%)	2 (2.9%)	
NYHA class				
I	19 (18.6%)	4 (30.8%)	15 (16.9%)	
II	30 (29.4%)	3 (23.1%)	27 (30.3%)	
III	45 (44.1%)	4 (30.8%)	41 (46.1%)	
IV	8 (7.8%)	2 (15.4%)	6 (6.7%)	
Diabetes mellitus	20 (19.8%)	3 (23.1%)	17 (19.3%)	1.00
Hypertension	48 (47.1%)	9 (69.2%)	39 (43.8%)	.22
Pulmonary disease	15 (15.0%)	1 (7.7%)	14 (16.1%)	.71
Neurological disease	5 (6.2%)	1 (8.3%)	4 (5.8%)	.56
RV function				
Good	48 (47.1%)	5 (38.5%)	43 (48.3%)	
Mildly impaired	13 (12.7%)	3 (23.1%)	10 (11.2%)	
Moderately impaired	31 (30.4%)	5 (38.5%)	26 (29.2%)	
Severely impaired	10 (9.8%)	0	10 (11.2%)	
Tricuspid regurgitation				
No	43 (47.3%)	6 (50.0%)	37 (46.8%)	
Mild	41 (45.1%)	5 (41.7%)	36 (45.6%)	
Moderate	5 (5.5%)	0	5 (6.3%)	
Severe	2 (2.2%)	1 (8.3%)	1 (1.3%)	
LVEF category				
Good (>50%)	75 (73.5%)	8 (61.5%)	67 (75.3%)	
Moderate (31%-50%)	25 (24.5%)	5 (38.5%)	20 (22.5%)	
Poor (<30%)	2 (2.0%)	0	2 (2.2%)	
Cardiogenic shock	1 (1.2%)	1 (9.1%)	0	
Preoperative ventilated	1 (1.1%)	1 (9.1%)	0	
IABP				
No	80 (89.9%)	9 (81.8%)	71 (91.0%)	
Pre-op	1 (1.1%)	1 (9.1%)	0	
Intra-op	6 (6.7%)	1 (9.1%)	5 (6.4%)	
Postop	2 (2.2%)	0	2 (2.6%)	
Intraoperative findings				
CPB	73 (71.6%)	8 (61.5%)	65 (73.0%)	.39
Cross-clamp time, minutes		85 ± 34	45 ± 41	<.01
CPB time, minutes		132 ± 48	100 ± 50	.09
Concomitant procedures				
CABG	11 (10.8%)	5 (38.5%)	6 (6.7%)	<.01
Valve	12 (11.8%)	4 (30.8%)	8 (9.0%)	.07
Postoperative findings				
Hospital LOS, days	16 ± 14	23 ± 20	15 ± 13	.05
ITU LOS, days	15 ± 85	91 ± 25	5 ± 8	<.01
Deep sternal wound infection	4 (3.9%)	0	4 (4.5%)	.99
Return to theatre	10 (9.8%)	1 (7.7%)	9 (10.1%)	1.00
Neurological dysfunction	7 (6.9%)	1 (7.7%)	6 (6.7%)	1.00
Haemofiltration/dialysis	1 (1.0%)	0	1 (1.1%)	1.00
Low cardiac output syndrome	46 (48.9%)	7 (58.3%)	39 (47.6%)	.70
RV failure	46 (48.9%)	7 (58.3%)	39 (47.6%)	.70
Respiratory	14 (15.9%)	3 (30.0%)	11 (14.1%)	.40
Liver dysfunction	28 (29.8%)	5 (41.7%)	23 (28.0%)	0.53
Recurrence	1 (1.1%)	0	1 (1.2%)	1.00
Postoperative mortality	7 (6.9%)	1 (8.3%)	6 (6.7%)	1.00
Follow-up outcomes				
NYHA class				
I	42 (47.2%)	2 (18.2%)	40 (51.3%)	
II	39 (43.8%)	7 (63.6%)	32 (41.0%)	
III	7 (7.9%)	2 (18.2%)	5 (6.4%)	
IV	1 (1.1%)	0	1 (1.3%)	
RV function				
Good	34 (39.0%)	3 (33.3%)	31 (39.7%)	
Mildly impaired	27 (31.0%)	2 (22.2%)	25 (32.1%)	
Moderately impaired	14 (16.1%)	2 (22.2%)	12 (15.4%)	
Severely impaired	12 (13.8%)	2 (22.2%)	10 (12.8%)	

Statistical significance indecated by (*P* < .05). Continuous variables are presented as mean ± SD (normally distributed data) or median (interquartile range) for non-normally distributed data; normality was assessed using the Shapiro-Wilk test. Between-group comparisons were performed using the independent-samples Student’s *t*-test or the Mann-Whitney *U*-test as appropriate. Categorical variables are reported as frequency (percentage) and compared using the chi-squared test or Fisher’s exact test.

Abbreviations: BMI, body mass index; CABG, coronary artery bypass grafting; CCS, Canadian Cardiovascular Society; CPB, cardiopulmonary bypass; IABP, intra-aortic balloon pump; ITU, intensive care unit; LVEF, left ventricular ejection fraction; NYHA, New York Heart Association; RV, right ventricle.

Patients in the partial pericardiectomy group were older (67 ± 11 vs 63 ± 12 years; *P* = .240) and had substantially higher operative risk as quantified by EuroSCORE II (7.2 ± 4.6 vs 4.9 ± 2.7; *P* = .010) and logistic EuroSCORE (14.5 ± 19.3 vs 5.6 ± 6.8; *P* < .001). Operative urgency differed significantly, with a higher proportion of urgent and emergency cases in the partial group (61.5% urgent; 15.4% emergency) compared with the total group (43.8% urgent; 4.5% emergency). Preoperative NYHA class distribution was broadly comparable: 51.9% of total pericardiectomy patients were in NYHA class III-IV versus 46.2% in the partial group. Preoperative RV function categories showed no statistically significant difference between the groups (*P* = .353).

Concomitant procedures were more frequent in the partial group (38.5% [5/13] vs 6.7% [6/89]; *P* < .001), predominantly CABG. One patient in each group required preoperative IABP; 2 partial-group patients had cardiogenic shock or required preoperative ventilation. TR was mild in the majority of both the groups (41.7% partial vs 45.6% total). Right heart catheterization was performed preoperatively in 43 of 89 total pericardiectomy patients (48.3%) and in 2 of 13 partial pericardiectomy patients (15.4%). In those undergoing RHC, mean pulmonary artery systolic pressure was 37.6 ± 8.6 mmHg in the total group and 40.1 ± 11.1 mmHg in the partial group. In the remaining patients, constrictive physiology was established by echocardiographic criteria (annulus paradoxus, respiratory variation in mitral E-velocity) and, where indicated, cardiac computed tomography; the decision to omit invasive haemodynamic assessment was at the discretion of the operating surgeon and cardiologist.

### Operative findings

Cardiopulmonary bypass was used in 73.0% of cases (65/89 total; 8/13 partial; *P* = .390). Cross-clamp time was longer after total pericardiectomy (85 ± 34 vs 45 ± 41 min; *P* < .001), reflecting greater extent of decortication; CPB duration did not differ significantly (132 ± 48 vs 100 ± 50 min; *P* = .090). Dense calcification and adhesions were encountered in both the groups.

### Postoperative outcomes

Early mortality was comparable (total 6.7% [6/89] vs partial 8.3% [1/13]; *P* = 1.00), consistent with published 30-day mortality of 5%-10%.^9^^,^^14^ Morbidity was broadly similar: low cardiac output syndrome (47.6% vs 58.3%; *P* = .700), RV failure (47.6% vs 58.3%; *P* = .700), liver dysfunction (28.0% vs 41.7%; *P* = .310). Recurrent constriction occurred in one total-group patient.

Despite the shorter cross-clamp time associated with partial resection, this did not translate into expedited postoperative recovery. Total hospital length of stay was significantly longer after partial pericardiectomy (23 ± 20 vs 15 ± 13 days; *P* = .050), as was ITU stay. This likely reflects the greater baseline morbidity of the partial pericardiectomy cohort combined with persistent haemodynamic impairment from incomplete decortication.

### Right ventricular function

**Figure 2.**
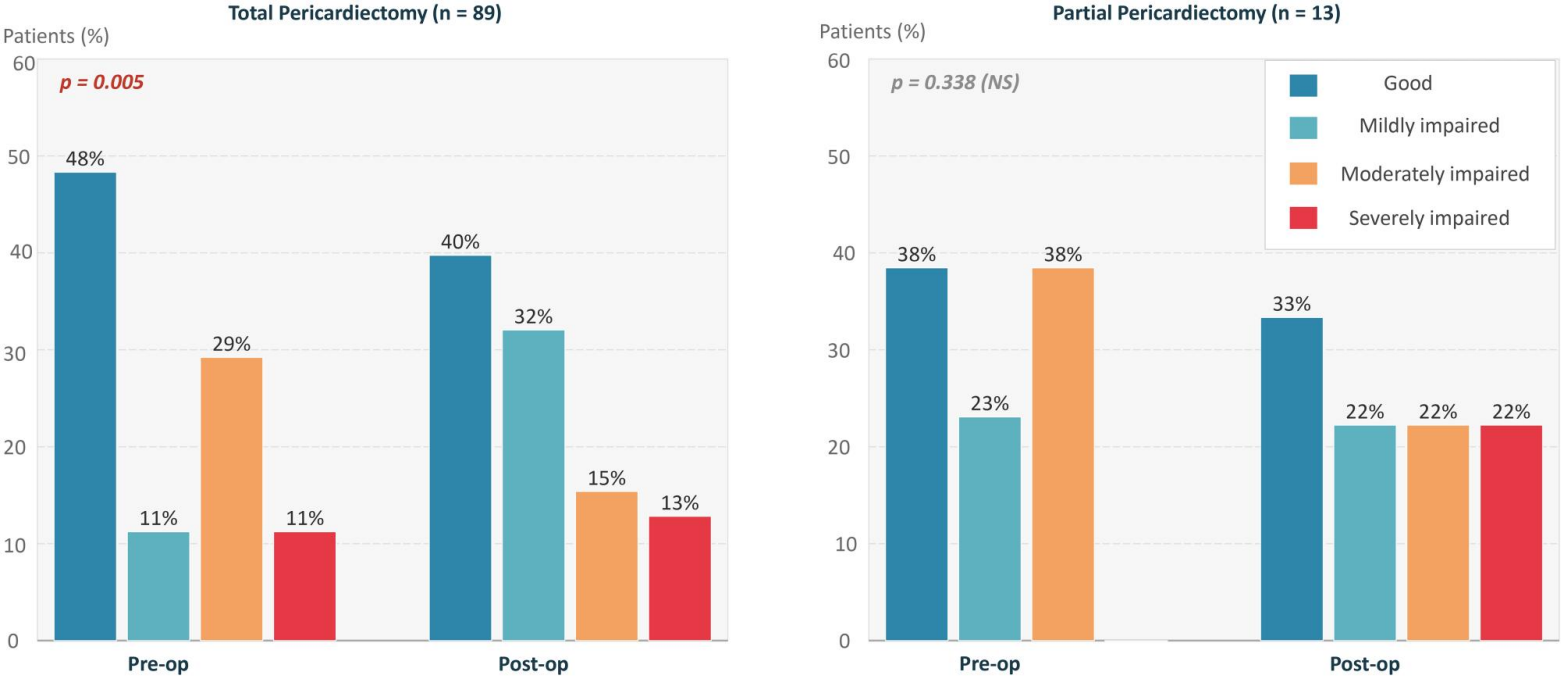
Right Ventricular (RV) Function Distribution Preoperatively and at Follow-Up, Stratified by Operative Extent. Total group (*n* = 89): significant shift in RV function (chi-squared for trend, *P* = .005) with increased mildly impaired function postoperatively, consistent with transient RV volume overload. Partial group (*n* = 13): no significant change (*P* = .338)

Changes in RV function are illustrated in **Figure 2**. The total group showed significant redistribution in RV function category (*P* = .005), with decreased “good” function (48.3%-39.7%) and increased “mildly impaired” function (11.2%-32.1%)—an expected transient dysfunction after abrupt release of chronic pericardial constraint.^15^ No significant change was seen in the partial group (*P* = .338), likely reflecting sample size.

### Long-term survival

**Figure 3.**
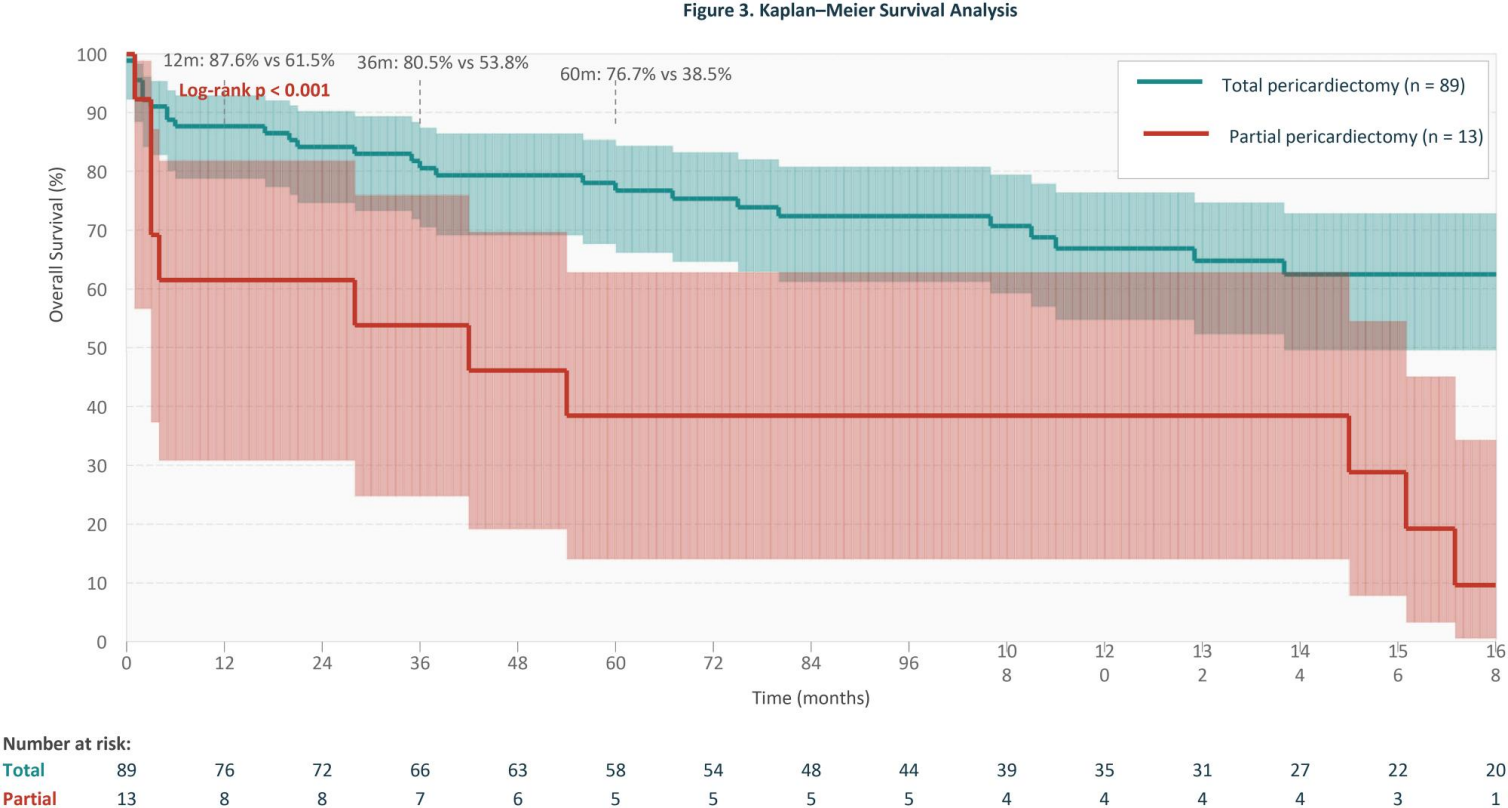
Kaplan-Meier Survival After Total Versus Partial Pericardiectomy, With 95% CI and Number-At-Risk. Log-rank *P* < .001. Estimated 1-, 3-, 5-year survival: 87.6%, 80.5%, 76.7% (total) Versus 61.5%, 53.8%, 38.5% (partial). Adjusted Cox HR 0.38 (95% CI 0.18-0.80; *P* = .011)

Kaplan-Meier survival analysis (**Figure  3**) demonstrated a highly significant difference in long-term survival favouring total pericardiectomy (log-rank *P* < .001). Estimated 1-, 3-, and 5-year survival was 87.6% (95% CI 78.8%-93.0%), 80.5% (95% CI 70.5%-87.4%), and 76.7% (95% CI 66.2%-84.3%) for the total group, versus 61.5%, 53.8%, and 38.5% for the partial group, respectively. On Cox proportional hazards regression, total pericardiectomy was associated with a crude HR for all-cause death of 0.30 (95% CI 0.15-0.60; *P* < .001).

### Functional status at follow-up

**Figure 4.**
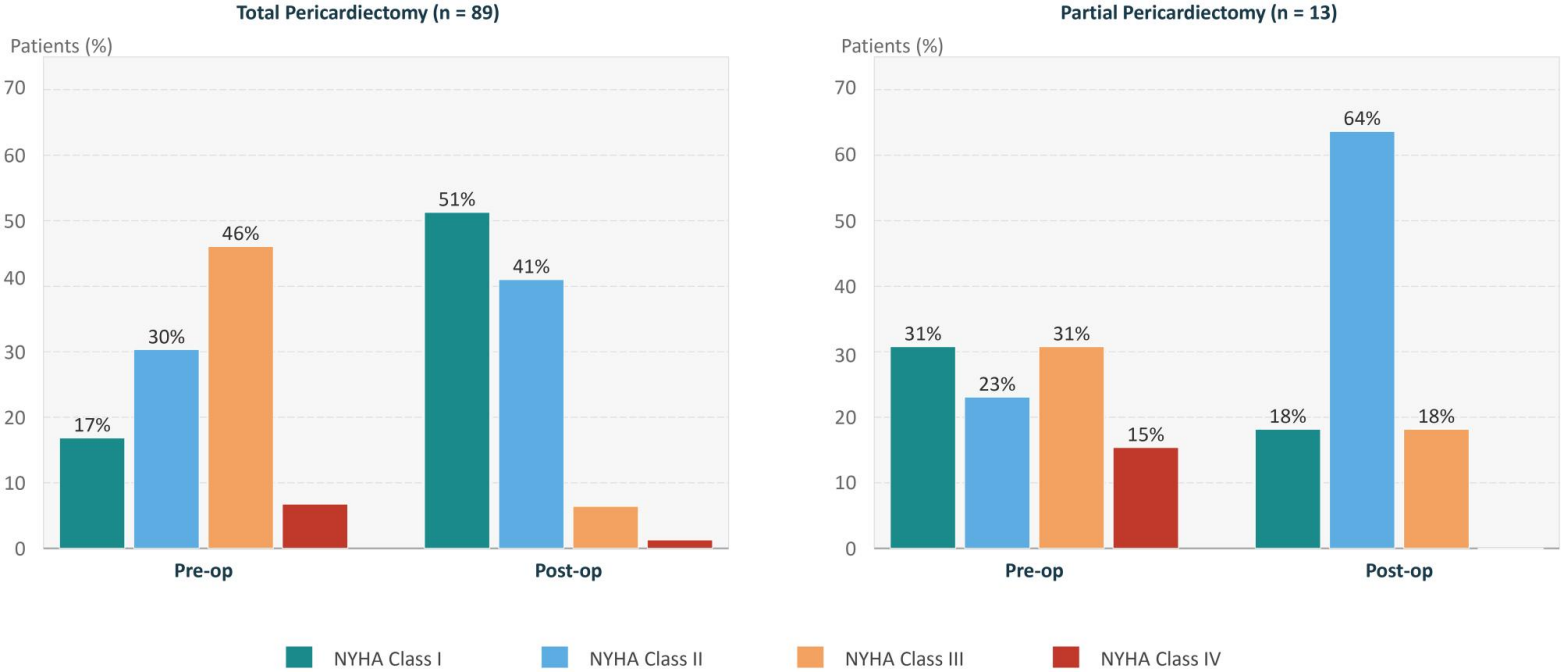
New York Heart Association (NYHA) Functional Class Distribution

New York Heart Association class at follow-up (**Figure  4**) showed substantial improvement in both the groups. In the total group, 51.3% reached class I and 41.0% class II; in the partial group, only 18.2% reached class I and 63.6% class II, with greater residual class III symptoms (18.2%).

## DISCUSSION

This 2-decade series of 102 consecutive pericardiectomies is one of the largest contemporary UK institutional experiences in CP. Three findings emerge: partial pericardiectomy was selected for a higher-risk cohort; despite this, it conferred no short-term recovery advantage and was associated with longer hospital stays; and long-term survival was markedly superior after total pericardiectomy, with a 33 percentage-point difference at 3 years.

### Extent of resection and survival

In the present series, the crude hazard ratio for all-cause mortality associated with total versus partial pericardiectomy was 0.30 (95% CI 0.15-0.60; *P* < .001), attenuating to an adjusted HR of 0.38 (95% CI 0.18-0.80; *P* = .011) after covariate adjustment for age, sex, and EuroSCORE II. This represents a 62% reduction in crude mortality risk and a 62% adjusted risk reduction associated with total resection. These findings align with Chowdhury et al.,^13^ who reported a 4.5-fold higher death risk with partial resection in 395 patients, and with European^16^ and Cleveland Clinic^17^ series confirming superior long-term survival after radical resection. The biological rationale is compelling: incomplete decortication leaves residual constriction, sustains venous congestion, and denies the RV the volume reserve needed for recovery.^18^

### The role of CPB

The role of CPB remains debated. Early data associating CPB with higher mortality likely reflected case complexity rather than bypass itself: the Cleveland Clinic series^17^ showed equivalent on- versus off-pump mortality (6.0% vs 4.9%; *P* = .580), and Mayo Clinic^9^ and Huang^12^ data support CPB as an enabler of complete posterior decortication, particularly where calcification is dense. In our series, 73.0% of total pericardiectomy cases were on-pump, consistent with selective CPB for cases requiring cross-clamp or concomitant procedures.

An alternative approach, bilateral sequential mini-thoracotomy, achieves lateral decortication through 2 small incisions without median sternotomy; it has been advocated for laterally predominant calcification or where repeat sternotomy carries prohibitive risk.^11^ However, it cannot access the anterior interventricular groove, diaphragmatic surface, or posterior pericardium, and is therefore unsuitable for total biventricular decortication. In our practice, median sternotomy with selective CPB has provided adequate exposure for total pericardiectomy in the majority of cases.

### Postoperative RV dysfunction

The shift towards mildly impaired RV function after total pericardiectomy (32.1% postoperative vs 11.2% preoperative; *P* = .0049) reflects abrupt transition from chronic pressure-volume constraint to unrestricted filling, exposing remodelled RV musculature to augmented preload. Busch et al.^14^ identified preoperative RV dilatation as an independent predictor of early mortality (OR 3.5; *P* = .040). Management relies on judicious fluid balance, targeted vasopressor-inotrope support, and IABP where appropriate.^10^

### Tricuspid regurgitation

Management of TR at pericardiectomy requires individualized judgement. TR in our cohort was predominantly mild (45.1%), and tricuspid intervention was not routine, consistent with the hypothesis that early adequate pericardiectomy permits regression of functional TR.^19^ However, Gongora et al.^20^ reported TR resolution in only 29% after pericardiectomy and showed that residual moderate-or-greater TR independently increased late mortality. We therefore endorse selective tricuspid repair when TR is moderate-or-greater, when anatomy precludes regression, or where additional operative risk is acceptable, consistent with 2015 ESC recommendations.^4^

### Aetiology and its impact on outcomes

Although not a primary end-point, aetiology is a powerful determinant of long-term outcomes after pericardiectomy.^3^ George et al.^3^ reported 5-year survival ranging from 79.8% (idiopathic) to 55.9% (postsurgical) to 11.0% (postradiation). Our cohort reflects the contemporary UK pattern, predominantly idiopathic or postcardiac surgery with smaller proportions of postradiation and tuberculous cases. Postradiation patients deserve particular mention owing to concurrent myocardial fibrosis, frequent need for concomitant valve surgery, and higher operative mortality.^21^ Avgerinos et al.^10^ reported 15-year survival of 78.3% after predominantly total pericardiectomy, with elevated bilirubin and creatinine as significant long-term mortality predictors.

### Functional recovery

Marked improvement in NYHA class was observed in both the groups, validating pericardiectomy as effective symptom relief. However, the higher proportion reaching NYHA class I after total pericardiectomy (51.3% vs 18.2%) reinforces that complete decortication—not merely palliation—is the therapeutic goal. Untreated CP carries up to 90% mortality,^22^ and postoperative outcomes are strongly influenced by preoperative functional status,^9^ supporting early referral and definitive total resection before advanced end-organ dysfunction. Our data support the ESC Class I recommendation for pericardiectomy in symptomatic CP.^4^

### Limitations

This study has important limitations. The retrospective single-centre design introduces selection and ascertainment biases. The small partial pericardiectomy cohort (*n* = 13) limits statistical power, and Cox adjustment for age, sex, and EuroSCORE II cannot fully account for residual unmeasured confounding. Right heart catheterization was not uniform (44.1% overall; 15.4% partial group), reflecting variation in preoperative workup. Aetiology-specific survival analysis could not be performed given sample size, and cause-specific mortality data were incomplete. The small size of the partial pericardiectomy cohort (*n* = 13) substantially limits statistical power for between-group comparisons, and the Cox regression analysis, while adjusting for key confounders (age, sex, EuroSCORE II), cannot fully account for all sources of selection bias inherent in the non-randomized allocation between groups. Critically, the partial pericardiectomy group carried a significantly higher EuroSCORE II, higher rates of urgent/emergency surgery, and more frequent concomitant procedures, all of which are independently associated with adverse outcomes; although the adjusted Cox regression partially addressed this confounding, residual unmeasured confounding cannot be excluded. Right heart catheterization was not uniformly performed (44.1% overall; 15.4% in the partial group), reflecting variation in preoperative workup driven by clinical urgency; the incomplete availability of invasive haemodynamic data represents an additional limitation. Aetiology-specific survival analysis could not be performed given sample size constraints. Finally, cause-specific mortality data were not systematically available for all patients, precluding analysis of disease-related versus non-disease-related late death.

## CONCLUSION

This 2-decade single-centre experience provides compelling evidence that total pericardiectomy, performed with selective use of CPB to facilitate complete biventricular decortication, is associated with significantly superior long-term survival and functional recovery compared with partial resection in patients with CP. On multivariable Cox regression adjusted for age, sex, and EuroSCORE II, total pericardiectomy was independently associated with a 62% reduction in the hazard of all-cause death (adjusted HR 0.38, 95% CI 0.18-0.80; *P* = .011). Despite greater complexity, total pericardiectomy did not worsen early mortality and was associated with shorter postoperative stay. These findings support total pericardiectomy as the standard of care and reinforce early surgical referral before advanced end-organ dysfunction precludes safe radical resection. Prospective multicentre registry studies are required to further define the role of CPB, timing of surgery, and indications for concomitant tricuspid repair.

## Data Availability

The data underlying this article are available in the article.

## ABBREVIATIONS

CABG: Coronary artery bypass grafting
CP: Constrictive pericarditis
CPB: Cardiopulmonary bypass
EuroSCORE: European System for Cardiac Operative Risk Evaluation
IABP: Intra-aortic balloon pump
LVEF: Left ventricular ejection fraction
NYHA: New York Heart Association
RV: Right ventricle
TR: Tricuspid regurgitation
TU: Intensive care unit
